# Exploring the lived experiences of drivers in facing floods caused by overflowing rivers and water channels

**Published:** 2024-10

**Authors:** Faeze Kobrai-Abkenar, Pooya Nematzad, Parand Pourghane

**Affiliations:** ^*a*^ Zeynab (P.B.U.H) School of Nursing and Midwifery, Guilan University of Medical Sciences, Rasht, Iran.; ^*b*^ Department of Nursing, Zeynab (P.B.U.H) School of Nursing and Midwifery, Guilan University of Medical Sciences, Rasht, Iran.

## Abstract

**Background:**

Flood is one of the common natural disasters all over the world that causes many direct and indirect damages, one of the most direct and terrible damages is death due to drowning. Unfortunately, these deaths occur in various forms and one of them is drowning in vehicles, including cars. Therefore, the researchers decided to investigate the experiences of drivers who had encountered floods caused by overflowing rivers and canals.

**Methods:**

The current research method is a qualitative approach. Initially, a variety of different sources were employed to find the related literature. The sources included web-based browsers of Science Direct, PubMed, Scopus, Elsevier and Google Scholar, SID, Irandoc, Magiran, and Iranmedex with a combination of keywords; “vehicles“, “driving“, “cars“, “drowning“, “flood“, “qualitative study“, and “experience“. The inclusion criteria were published studies between July 2000 and July 2024., having relation to the purpose of the study, being in English or Farsi language, and being available in full text. The texts were examined by two researchers independently. Finally, among the 24 articles, 4 articles were selected and analyzed.

**Results:**

After examining the findings of 4 articles related to the lived experiences of drivers, what affects the decisions of drivers when faced with floods and leads to their entering or not entering floods can be divided into two categories: factors affecting decision-making and effective factors on risk assessment.

**Conclusions:**

The results of the present study can enhance understanding of drivers’ behavioral decisions during floods. This, in turn, will aid in developing preventive interventions and save human lives lost in the waterways during floods. Such interventions include designing and developing programs to promote safe driving behavior during floods, taking into account social, demographic, and environmental factors.

**Keywords:**

Motor vehicles, Automobile driving, Drowning, Floods

